# Coutilization of D-Glucose, D-Xylose, and L-Arabinose in* Saccharomyces cerevisiae* by Coexpressing the Metabolic Pathways and Evolutionary Engineering

**DOI:** 10.1155/2017/5318232

**Published:** 2017-03-26

**Authors:** Chengqiang Wang, Jianzhi Zhao, Chenxi Qiu, Shihao Wang, Yu Shen, Binghai Du, Yanqin Ding, Xiaoming Bao

**Affiliations:** ^1^College of Life Sciences and Shandong Key Laboratory of Agricultural Microbiology and National Engineering Laboratory for Efficient Utilization of Soil and Fertilizer Resources, Shandong Agricultural University, Tai'an, China; ^2^The State Key Laboratory of Microbial Technology, Shandong University, Jinan, China

## Abstract

Efficient and cost-effective fuel ethanol production from lignocellulosic materials requires simultaneous cofermentation of all hydrolyzed sugars, mainly including D-glucose, D-xylose, and L-arabinose.* Saccharomyces cerevisiae* is a traditional D-glucose fermenting strain and could utilize D-xylose and L-arabinose after introducing the initial metabolic pathways. The efficiency and simultaneous coutilization of the two pentoses and D-glucose for ethanol production in* S. cerevisiae* still need to be optimized. Previously, we constructed an L-arabinose-utilizing* S. cerevisiae* BSW3AP. In this study, we further introduced the XI and XR-XDH metabolic pathways of D-xylose into BSW3AP to obtain D-glucose, D-xylose, and L-arabinose cofermenting strain. Benefits of evolutionary engineering: the resulting strain BSW4XA3 displayed a simultaneous coutilization of D-xylose and L-arabinose with similar consumption rates, and the D-glucose metabolic capacity was not decreased. After 120 h of fermentation on mixed D-glucose, D-xylose, and L-arabinose, BSW4XA3 consumed 24% more amounts of pentoses and the ethanol yield of mixed sugars was increased by 30% than that of BSW3AP. The resulting strain BSW4XA3 was a useful chassis for further enhancing the coutilization efficiency of mixed sugars for bioethanol production.

## 1. Introduction

For the decreasing of fossil energy resources, fuel ethanol was proposed as an important renewable energy and the requirement of bioethanol production is more than ever [1, 2]. Future large-scale production of fuel ethanol will most certainly be based on lignocellulosic materials, which are the most abundant stock in the world [3]. In industry, economical and efficient fuel ethanol production process from lignocellulosic materials requires cofermentation of all hydrolyzed hexoses and pentoses [4]. Moreover, D-xylose and L-arabinose are the most abundant pentoses, which should be utilized [5].

Constructing microorganisms which are capable of fermenting mixed sugars simultaneously is the main challenge in optimizing the production and application of biofuels [6].* Saccharomyces cerevisiae* is a traditional strain for fermenting ethanol, which is robust, safe, and suitable for fermenting lignocellulosic hydrolysates. Wild-type* S. cerevisiae* cannot utilize pentose because of the lack of initial metabolic pathways [7]. By expressing heterologous D-xylose and L-arabinose metabolic pathways,* S. cerevisiae* could obtain the metabolic capacity [8, 9]; however the efficiency still needs to be further improved.

The main strategies for constructing D-xylose-utilizing* S. cerevisiae* include two pathways [10, 11]. One is XR-XDH pathway, which contains D-xylose reductase (XR) and xylitol dehydrogenase (XDH), and converts D-xylose to xylulose in* S. cerevisiae* [12, 13]. Because of the cofactor imbalance in this pathway, the accumulation of byproduct xylitol was the main bottleneck to be solved [14]. Another is XI pathway, which only needs to introduce one D-xylose isomerase (XI), that directly converts D-xylose to xylulose [15, 16]. In this strategy, the activity of XI still needs to be increased [17, 18]. The xylulose from both pathways could then be phosphorylated to xylulose-5-P by endogenous xylulokinase. Xylulose-5-P was further entered into the endogenous pentose phosphate pathway (PPP) to produce ethanol. There are also two main L-arabinose metabolic pathways from fungi or bacteria, which are both candidates for constructing L-arabinose-metabolic yeasts. L-Arabinose could be converted to D-xylulose-5-phosphate through either fungal or bacterial pathway, and then D-xylulose-5-phosphate enters into PPP. The fungal pathway needs five enzymes including aldose reductase (AR), L-arabinitol-4-dehydrogenase (LAD), L-xylulose reductase (LXR), D-xylulose reductase (XDH), and xylulokinase (XK) [19–21]. In addition, this pathway contained two reduction reactions which utilize NADPH, two oxidation reactions which generate NADH, and a kinase reaction. The bacterial pathway contains only three initial enzymes including L-arabinose isomerase (AI), L-ribulokinase (RK), and L-ribulose-5-P-4-epimerase (RPE), which do not require any cofactor [22–24].

Coutilization of D-xylose and L-arabinose could be obtained by combining expression of their metabolic pathways. The first attempt is coexpression of the XR-XDH pathway of D-xylose and the bacterial pathway of L-arabinose [25, 26]. The resulting strains could successfully coutilize of D-xylose and L-arabinose with D-glucose; however, a large amount of L-arabinose was converted to byproduct L-arabitol due to the aldose reductase activity of XR. The D-xylose XI pathway and the bacterial pathway of L-arabinose were then carried out to ignore the effect of XR [24, 27]. The resulting strains could coutilize D-xylose and L-arabinose with D-glucose to produce ethanol well and significantly decreased the byproducts. In this case, the simultaneous utilization capacity of strains to convert D-glucose, D-xylose, and L-arabinose to bioethanol should be further improved. Due to the fungal pathway of L-arabinose metabolism containing the metabolic enzymes for D-xylose utilization, the resulting strains were also able to utilize D-xylose to produce ethanol. The capacity to metabolize mixed sugars of this pathway was also studied in* S. cerevisiae* [20, 28]. In summary, the studies for simultaneous utilization of pentoses with D-glucose still need to be further performed for high efficiency.

In the present study, we report a different strategy to construct D-glucose, D-xylose, and L-arabinose cofermenting* S. cerevisiae*. The XI and XR-XDH metabolic pathways were both introduced into the L-arabinose utilizing* S. cerevisiae* BSW3AP to obtain D-xylose and L-arabinose coutilization capacity. Evolutionary engineering was further used to improve the pentose metabolic efficiency. The sugar utilization of the resulting strain was tested on mixed D-glucose, D-xylose, and L-arabinose and the advantages of this strain were also discussed.

## 2. Materials and Methods

### 2.1. Plasmid and Strain Construction

**Figure 1:**
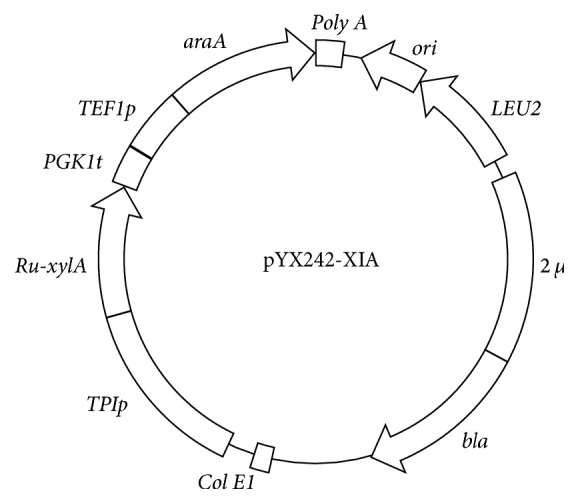
The physical map of plasmid pYX242-XIA.

The Ru-*xylA* fragments of D-xylose isomerase (XI) were cloned from plasmid pJX7 [15] and then inserted into pYX242-*TEF1araA* [29] between sites* Eco*R I and* Nco* I using Gibson assembly [30] to obtain pYX242-XIA (Figure 1).* Escherichia coli* DH5*α* was used for plasmid amplification, subcloning, and gene sequencing.

The formerly obtained L-arabinose fermentation strain BSW3AP [29] was used as the chassis. The yeast transformation was conducted by the conventional lithium acetate transformation method [31]. The episomal plasmid pYX242-*TEF1araA* of BSW3AP was firstly lost by long time cultivation adding enough leucine and then resulting strain BSW4AP. The plasmid pYX242-XIA was then transferred into BSW4AP to obtain strain BSW4XA1. The plasmid pYMIK-xy127 [32] containing the XR-XDH pathway of D-xylose was digested by restriction endonuclease* Hpa* I and then integrated into the chromosome of BSW4XA1 to obtain BSW4XA2. After an extensive evolutionary engineering on D-xylose, the evolved strain BSW4XA3 exhibited better sugar coutilizing capacity.

*S. cerevisiae* strains and plasmids used in this study are listed in Table 1. The primers used in this study are summarized in Table 2.

### 2.2. Media and Incubation

The yeast synthetic complete (SC) medium contains 1.7 g L^−1^ yeast nitrogen base (YNB, Sangon, China), 5 g L^−1^ ammonium sulfate (Sangon, China), and the complete supplement mixture 0.77 g L^−1^ CSM-URA or 0.67 g L^−1^ CSM-LEU-URA (MP Biomedicals, Solon, OH), and the needed carbon sources (D-glucose, D-xylose or L-arabinose) were used for cultivation of constructed yeasts and maintaining the required plasmids. About 200 *μ*g mL^−1^ G418 was supplied in the culture when needed. Plasmids were amplified in* E. coli* strain DH5*α* (TransGen Biotech, China) growing on Luria-Bertani (LB) medium with 200 *μ*g mL^−1^ ampicillin.

To cultivate BSW3AP derived strains by batch cultivation, the single colonies were preincubated two times in SC medium containing 20 g L^−1^ D-glucose for 24 h and 12 h, respectively. After that, cells were collected and used for batch cultivation in the same SC medium containing the needed carbon sources (D-glucose, D-xylose, or L-arabinose). All the batch incubations of yeasts were performed in 40 mL culture for aerobiotic cultivation or oxygen-limited fermentation at 30°C, 200 r min^−1^. All the cultivations of* E. coli* strains were performed at 37°C, 200 r min^−1^.

### 2.3. Growth Measurement

The culture optical density (OD_600_) was measured by a BioPhotometer plus (Eppendorf, Germany) to obtain the growth curves. The growth capacities of strains were determined by the exponential growth rates [33], which were analyzed by the linear regression coefficients of ln⁡OD_600_ versus growth hours from the growth curves [34]. The dry cell weight (DCW) of the strains was calculated using the formula of dry weight (mg mL^−1^) which is equal to 0.266 × OD_600_ − 0.0762 [29].

### 2.4. The Analysis of Metabolites

The concentrations of D-glucose, D-xylose, xylitol, L-arabinose, L-arabitol, and ethanol were determined in the supernatant of filtered samples from oxygen-limited batch cultivation. The high performance liquid chromatography (HPLC) prominence LC-20A (Shimadzu, Japan) with a refractive index detector RID-10A (Shimadzu, Japan) and an Aminex HPX-87P ion exchange column (Bio-Rad, USA) was used to determine the concentration of the above chemicals at 80°C with a mobile phase of water at a flow rate of 0.6 mL min^−1^, as reported [25, 29].

## 3. Results

### 3.1. Coexpressing of D-Xylose Metabolic Pathways in BSW3AP

**Figure 2:**
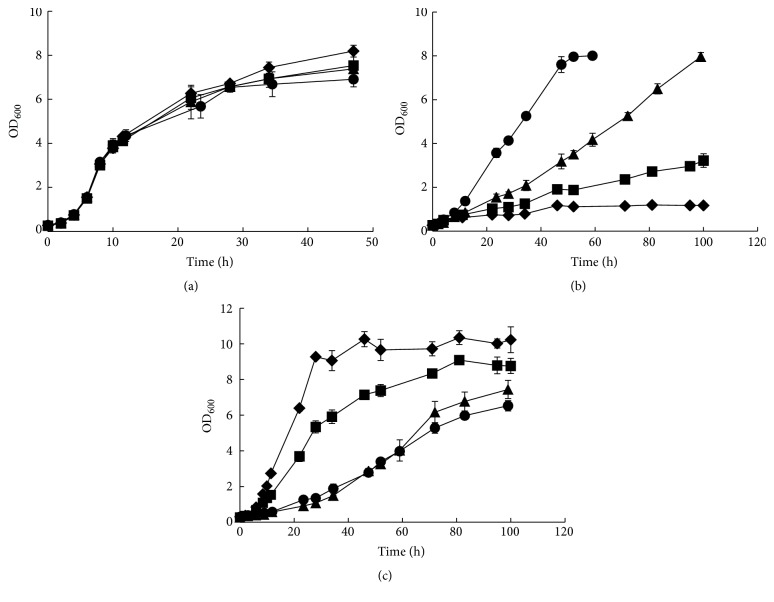
The growth curves of strains BSW3AP (◆), BSW4XA1 (■), BSW4XA2 (▲), and BSW4XA3 (●) on 20 g L^−1^ D-glucose (a), D-xylose (b), and L-arabinose (c), respectively. The strains were preincubated in SC-Leu-Ura medium containing 20 g L^−1^ D-glucose for 24 h and then transferred using 10 g L^−1^ D-glucose and 20 g L^−1^ L-arabinose as the carbon sources to incubate for 24 h. The cells were then collected and used for aerobiotic batch cultivation in 40 mL SC-Leu-Ura medium with 20 g L^−1^ D-glucose, D-xylose, or L-arabinose at 30°C, 200 r min^−1^, and the initial OD_600_ was 0.3. The data are the averages of three independent tests.

The D-xylose isomerase (XI) Ru-*xylA* fragments were formerly cloned from bovine rumen metagenome and tested to have higher enzyme activities than other tested ones in* S. cerevisiae* by our group [15]. So, this gene was overexpressed into the episomal plasmid pYX242-*TEF1araA* to construct plasmid pYX242-XIA (Figure 1). The formerly obtained L-arabinose fermentation strain BSW3AP [29] was firstly losing the plasmid by long time cultivation with enough leucine to obtain strain BSW4AP. The plasmid pYX242-XIA was then transferred into strain BSW4AP to obtain strain BSW4XA1, which obtained the D-xylose metabolic capacity and could slowly grow on D-xylose as sole carbon source with the maximum specific growth rate (*μ*_max_) 0.035 h^−1^ (Figure 2(b)). However, the D-xylose metabolic capacity of BSW4XA1 was not high enough and not improved after long time evolutionary engineering (data not shown). Furthermore, the D-xylose reductase (XR), xylitol dehydrogenase (XDH), and xylulokinase (XK) of XR-XDH pathway in the plasmid pYMIK-xy127 [32] were then integrated into the chromosome of BSW4XA1 to obtain BSW4XA2 and *μ*_max_ on D-xylose was further increased up to 0.047 h^−1^ (Figure 2(b)). The growth capacities of BSW4XA1 and BSW4XA2 on D-glucose were not affected (Figure 2(a)), but *μ*_max_ on L-arabinose was otherwise decreased from 0.2 h^−1^ to 0.158 h^−1^ and 0.047 h^−1^, respectively (Figure 2(c)).

### 3.2. Improving D-Xylose Metabolic Capacity by Evolutionary Engineering

**Figure 3:**
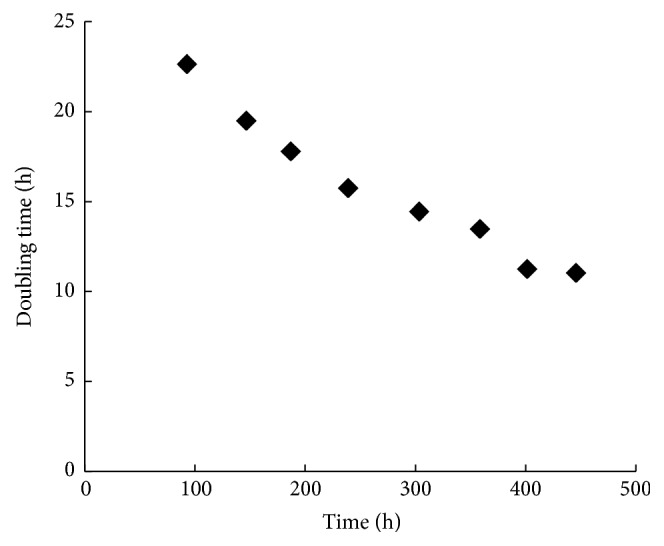
Adaptive cultivation of strain BSW4XA2 on D-xylose. The precultured BSW4XA2 was conducted in a series of batch cultures on 20 g L^−1^ D-xylose in air-limited condition until the doubling time is stable.

Evolutionary engineering was a useful tool for improving recombinant strains to growth on sugars [27, 35]. The strategy of evolutionary engineering was used here to evolve BSW4XA2 on D-xylose batch culture in air-limited condition. After approximately eight times of transfer, the estimated doubling time was stable at about 11 h (Figure 3). Selected on several D-glucose or D-xylose plates, a big colony was selected out and the ability of D-xylose efficient utilization remained after three times transfer on D-glucose. The selected strain was named BSW4XA3 and *μ*_max_ on D-xylose was further increased to 0.062 h^−1^ (Figure 2(b)). It is noteworthy that the growth capacity of it on L-arabinose was not distinctly decreased (Figure 2(c)) and *μ*_max_ was 0.046 h^−1^. The growth capacity of it on D-glucose was also not decreased (Figure 2(a)).

### 3.3. Coutilization of D-Glucose, D-Xylose, and L-Arabinose for Ethanol Production

**Figure 4:**
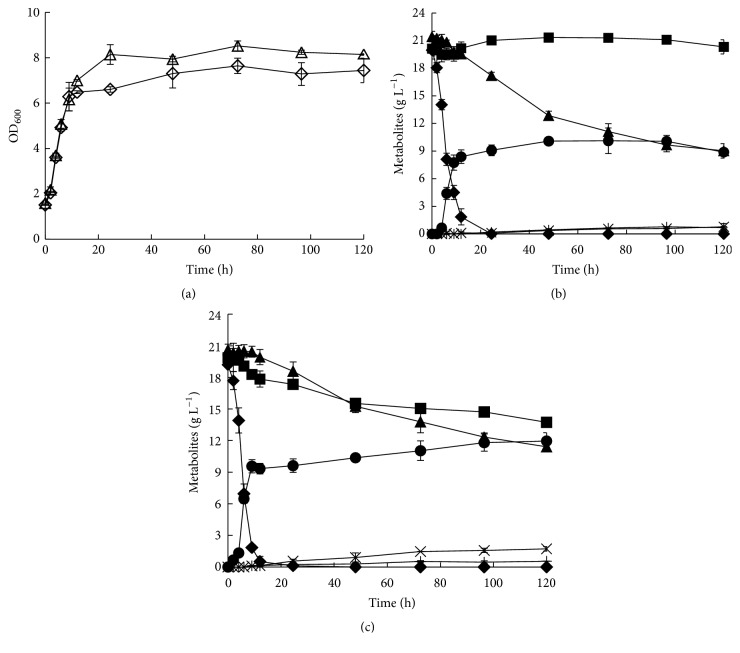
The metabolic capacities of BSW4XA3 and BSW3AP on mixed sugars of D-glucose, D-xylose, and L-arabinose. (a) The growth curves of strains BSW3AP (△) and BSW4XA3 (⋄). (b) The sugar utilization and sugar alcohols production of BSW3AP. (c) The sugar utilization and sugar alcohols production of BSW4XA3. D-Glucose (◆), D-xylose (■), L-arabinose (▲), ethanol (●), L-arabitol (**×**), and xylitol (**+**). The strains were preincubated in SC-Leu-Ura medium containing 20 g L^−1^ D-glucose for 24 h and then transferred using 20 g L^−1^ D-glucose, 20 g L^−1^ D-xylose, and 20 g L^−1^ L-arabinose as the carbon sources to incubate for 24 h. The cells were then collected and used for fermentation in 40 mL SC-Leu-Ura medium with 20 g L^−1^ D-glucose, 20 g L^−1^ D-xylose, and 20 g L^−1^ L-arabinose at 30°C, 200 r min^−1^, and the initial OD_600_ was 1.5. The data are the averages of three independent tests.

To investigate the capability of strain BSW4XA3 to utilize the mixed sugars of D-glucose, D-xylose, and L-arabinose, an oxygen-limited fermentation of BSW4XA3 and BSW3AP on 20 g L^−1^ D-glucose, 20 g L^−1^ D-xylose, and 20 g L^−1^ L-arabinose was performed (Figure 4 and Table 3). The strains BSW4XA3 and BSW3AP well grow on the mixed sugars with the maximum specific growth rates (*μ*_max_) of 0.17 h^−1^ and 0.16 h^−1^, respectively. The maximum OD_600_ of BSW4XA3 is lower than BSW3AP by about 10% (Figure 4(a)), which might indicate that more carbon sources can flow into the ethanol biosynthesis pathway instead of biomass synthesis in BSW4XA3. The strains BSW4XA3 and BSW3AP have similar D-glucose metabolic capacity, with the consumption rates 1.89 g h^−1^ g^−1^ DCW and 1.84 g h^−1^ g^−1^ DCW, respectively. After 6 h of fermentation, the strains started to prominently consume L-arabinose or D-xylose. BSW3AP could consume L-arabinose with a consumption rate of 0.1 g h^−1^ g^−1^ DCW and 12.42 g L^−1^ L-arabinose was consumed after 120 h of fermentation. During the same time, the L-arabinose consumption rate of BSW4XA3 was decreased to 0.059 g h^−1^ g^−1^ DCW and 9.22 g L^−1^ L-arabinose was consumed. BSW4XA3 successfully obtained the D-xylose metabolic capacity with a consumption rate of 0.055 g h^−1^ g^−1^ DCW to consume 6.14 g L^−1^ D-xylose after 120 h of fermentation. The consumption rates of L-arabinose and D-xylose were similar for BSW4XA3, which could utilize L-arabinose and D-xylose simultaneously. For ethanol productivity, the ethanol yield of BSW4XA3 from mixed sugars was higher than that of BSW3AP. After 120 h of fermentation, BSW4XA3 totally produced 12 g ethanol at an ethanol yield of 0.35 g_ethanol_ g_consumed sugars_^−1^; however, BSW3AP only produced 8.9 g ethanol at an ethanol yield of 0.27 g_ethanol_ g_consumed sugars_^−1^. In addition, the byproducts L-arabitol or xylitol of the two strains were little accumulated. The L-arabitol productivities of BSW3AP and BSW4XA3 were 0.78 g L^−1^ and 1.72 g L^−1^, respectively. Meanwhile, the xylitol productivities of BSW3AP and BSW4XA3 were 0.69 g L^−1^ and 0.55 g L^−1^, respectively.

## 4. Discussion

The complete and simultaneous conversion of total sugars from lignocellulosic materials is important for cost-effective bioethanol production [6]. Except D-glucose, the main contents are pentoses D-xylose and L-arabinose. Some studies had been focused on pentose metabolism to obtain* S. cerevisiae* strains owing to pentoses metabolic capacity, but the efficiencies remain suboptimal [11].

Our group formerly obtained an efficient L-arabinose fermentation strain BSW3AP [29], and in this study we used it as a chassis to introduce two D-xylose metabolic pathways. The D-xylose isomerase (XI) Ru-*xylA* fragments, which were formerly cloned from bovine rumen metagenome by our group [15], were firstly introduced. After further coexpression of the XR-XDH pathway in the chromosome and evolutionary engineering on D-xylose, the D-xylose metabolic capacities were gradually increased. We finally selected a strain BSW4XA3, which could coutilize mixed sugars.

The former coutilization studies of D-xylose and L-arabinose paid more attention to metabolizing D-xylose first, and the resulting strains somehow presented the gradual utilization of D-glucose, D-xylose, and L-arabinose [20, 28]. The coutilization efficiencies were not high enough. Now, we converse the strategy, introducing two D-xylose metabolic pathways to an L-arabinose fermenting strain. The resulting strain BSW4XA3 well obtained the D-xylose metabolic capacity. BSW4XA3 also presented good simultaneous conversion of D-xylose and L-arabinose to bioethanol with similar consumption rates, and the D-glucose metabolic capacity was not affected. By introducing two D-xylose metabolic pathways and evolutionary engineering, the L-arabinose metabolic capacity of BSW4XA3 was decreased which might be due to the same downstream pathway of the two pentoses from intermediate xylulose 5-phosphate [20]. In strain BSW3AP, the metabolic flux in the pentose downstream pathway was only contributed by L-arabinose. However, a part of the metabolic flux was occupied by D-xylose in BSW4XA3, which might reduce the L-arabinose metabolic efficiency to some extent. Although the metabolic flux of L-arabinose was decreased, the total metabolic flux of pentoses was increased by 24%. It was reported that XR could convert a large amount of L-arabinose to L-arabitol [25, 26], but our results showed that BSW4XA3 metabolized 9.22 g L^−1^ L-arabinose to only produce 0.94 g L^−1^ more L-arabitol than BSW3AP, which meant that only 18% L-arabinose was metabolized to L-arabitol. The successful decrease of L-arabitol might be the reason that we used the L-arabinose metabolic strain as the host cell to keep a high L-arabinose metabolic capacity. Meanwhile, the xylitol production was less because the XI pathway played a key regulation function and also might be the expression level of XR which was within an appropriate scope. More D-xylose led to producing ethanol and the ethanol yield was increased by 30% compared with BSW3AP. Former reported results and our results all showed that the pentose metabolic capacity was prominently lower than that of D-glucose due to D-glucose-inhibition effect. To alleviate the phenomenon, the pentose metabolic flux could be further improved and a pentose specific transporter without inhibition by D-glucose might also be further needed. The resulting strain BSW4XA3 in this study was an important basis for further improving the coutilization efficiency of mixed sugars.

## 5. Conclusions

The complete and simultaneous conversion of total sugars from lignocellulosic materials is important for cost-effective bioethanol production. Using a formally obtained and efficient L-arabinose fermentation strain as the chassis cell, we successfully introduced two D-xylose metabolic pathways. The resulting strain presented good ability for simultaneous conversion of D-xylose and L-arabinose to bioethanol; meanwhile, the D-glucose metabolic capacity was not affected. Furthermore, the total pentose fermentation amounts and bioethanol productivity were also significantly increased. Our present work provides a basis for further improving the coutilization efficiency of mixed sugars in actual bioethanol production process of lignocellulosic materials.

## Figures and Tables

**Table 1:** *S. cerevisiae* strains and plasmids used in this work.

	Genotype/properties	Source/reference
Strain		
BSW3AP	CEN.PK102-3A derivative, *gre3*(−241, +338):: *TPI1p–RKI1–RKI1t–PGK1p–TAL1–TAL1t–FBA1p–TKL1–TKL1t–ADH1p–RPE1–RPE1t–loxP*, {YIp5-ara, pYX2422-*TEF1araA*}, selected for growth on L-arabinose	[29]
BSW4AP	BSW3AP derivative, discarding plasmid pYX2422-*TEF1araA*	Present work
BSW4XA1	BSW4AP derivative, {pYX242-XIA}	Present work
BSW4XA2	BSW4XA1 derivative, {pYX242-XIA, pYMIK-xy127}	Present work
BSW4XA3	BSW4XA2 derivative, selected for growth on D-xylose	Present work
Plasmid		
pYX2422-*TEF1araA*	pYX242*-PGK1t*-*TEF1p*-*araA*	[29]
pYX242-XIA	pYX242*-Ru-xylA-PGK1t-TEF1p-araA*	Present work
pYMIK-xy127	Integration plasmid, *KanMX4*, *ADH1p-XYL1-ADH1t*, *PGK1p-XYL2-PGK1t*, *PGK1p-XKS1-PGK1t*	[32]

**Table 2:** The main DNA primers used in this work.

Primers	Sequence (5′-3′)	Purpose
One-XI up	GCTTAAATCTATAACTACAAAAAACACATACAGGAATTCATGGCAAAAGAATATTTTCC	Cloning Ru-*xylA* genes
One-XI down	TAGAGACATGGGAGATCCTAGCTAGCTAGATCCATGGTTATTTGCAGTGGAGGGCGACG	

pYX242-ce-F	GGAGTTTAGTGAACTTGCAAC	To validate or sequence the plasmid pYX242
pYX242-ce-R	CGACTCACTATAGGGCGAATTG	
PGKt-pYX2422-R	ATACGCTGAACCCGAACATAG	

**Table 3:** Physiological parameters of BSW4XA3 and BSW3AP fermentation on mixed sugars of 20 g L^−1^ D-glucose, 20 g L^−1^ D-xylose, and 20 g L^−1^ L-arabinose.

Strain	*μ*_max_^a^ (h^−1^)	Consumed sugars in 120 h (g L^−1^)		Sugar consumption rate (g h^−1^ g^−1^ DCW)			Ethanol production in 120 h (g L^−1^)	Ethanol yield^b^ (g_ethanol_ g_consumed sugars_^−1^)
		D-Xylose	L-Arabinose	D-Glucose	D-Xylose	L-Arabinose		
BSW3AP	0.16	0.00	12.42	1.84	0.00	0.10	8.89	0.27
BSW4XA3	0.17	6.14	9.22	1.89	0.055	0.059	11.95	0.35

The data are the averages of three independent tests.

^a^The maximum specific growth rate.

^b^Ethanol yield on all consumed sugars.
